# Worenine Prevents Solar Ultraviolet–Induced Sunburn by Inhibiting JNK2

**DOI:** 10.3389/fphar.2022.881042

**Published:** 2022-07-22

**Authors:** Juanjuan Xiao, Hui Lu, Tengfei Ma, Xiaofang Ni, Teding Chang, Man Liu, Nijie Li, Peijiang Lu, Changshu Ke, Qin Tian, Ling Zou, Fei Wang, Wei Wang, Lu Zhang, Ping Yuan, Lin Liu, Jianmin Zhang, Fei Shi, Qiuhong Duan, Feng Zhu

**Affiliations:** ^1^ Cancer Research Institute, The Affiliated Hospital of Guilin Medical University, Guilin, China; ^2^ Guangxi Health Commission Key Laboratory of Novel Onco-Kinases in Target Therapy, The Affiliated Hospital of Guilin Medical University, Guilin, China; ^3^ Wuhan Children’s Hospital (Wuhan Maternal and Child Healthcare Hospital), Tongji Medical College, Huazhong University of Science and Technology, Wuhan, China; ^4^ Department of Biochemistry and Molecular Biology, School of Basic Medicine, Tongji Medical College, Huazhong University of Science and Technology, Wuhan, China; ^5^ Second Clinical College, Tongji Medical College, Huazhong University of Science and Technology, Wuhan, China; ^6^ Department of Pathology, Tongji Hospital, Tongji Medical College, Huazhong University of Science and Technology, Wuhan, China; ^7^ Department of Dermatology, The General Hospital of Air Force, Beijing, China; ^8^ Guangxi Key Laboratory of Molecular Medicine in Liver Injury and Repair, The Affiliated Hospital of Guilin Medical University, Guilin, China

**Keywords:** TCM, traditional Chinese medicine, worenine, JNK2, SUV, sunburn

## Abstract

Excessive solar ultraviolet (SUV) radiation often causes dermatitis, photoaging, and even skin cancer. In the pathological processes of SUV-induced sunburn, JNK is activated by phosphorylation, and it in turn phosphorylates its downstream transcription factors, such as ATF2 and c-jun. The transcription factors further regulate the expression of pro-inflammatory genes, such as IL-6 and TNF-α, which ultimately leads to dermatitis. Therefore, inhibiting JNK may be a strategy to prevent dermatitis. In this study, we screened for worenine as a potential drug candidate for inhibiting sunburn. We determined that worenine inhibited the JNK-ATF2/c-jun signaling pathway and the secretion of IL-6 and TNF-α in cell culture and *in vivo*, confirming the role of worenine in inhibiting sunburn. Furthermore, we determined that worenine bound and inhibited JNK2 activity *in vitro* through the MST, kinase, and *in vitro* kinase assays. Therefore, worenine might be a promising drug candidate for the prevention and treatment of SUV-induced sunburn.

## Introduction

Excessive solar ultraviolet (SUV) radiation often causes a series of skin lesions, such as hyperpigmentation, dermatitis, photoaging, and even skin cancer (Del Bino et al., 2018; Sample and He, 2018; Sharma et al., 2018). UVA and UVB are important causes of sunburn and are classified as primary carcinogens by the International Agency for Research on Cancer (Mohania et al., 2017; Mullenders, 2018). UVA and UVB directly lead to DNA structural damage through photochemical action (Goto et al., 2015; Ikehata et al., 2018). In addition, UVA can also indirectly cause oxidative damage to DNA by generating ROS and RNS (Nikitaki et al., 2015; Cadet and Douki, 2018). DNA damage further leads to gene mutations and gene expression changes. For example, it directly causes the upregulation of inflammatory cytokines such as IL-6 and TNF-α, thereby inducing inflammation, photoaging, and skin cancer (Sabat et al., 2019). Therefore, the development of drugs for the prevention and treatment of sunburn is particularly important.

JNK, a stress-activated protein kinase (SAPK), is a member of the MAPK family (Dérijard et al., 1994; Kyriakis et al., 1994; Nogueiras and Sabio, 2021). JNK activation responds to cell pressure, such as SUV radiation (Matsushita et al., 2020). In the pathological processes of SUV-induced sunburn, JNK is activated by phosphorylation of MKK4 and MKK7 (Syc-Mazurek et al., 2018; Park et al., 2019). JNK in turn phosphorylates its downstream transcription factors, such as ATF2 and c-jun (Zeke et al., 2016). Transcription factors further regulate the expression of pro-inflammatory genes, such as IL-6, TNF-α, and other cytokines. Ultimately, this leads to inflammation, oxidative stress, and photoaging (Phuagkhaopong et al., 2017). The increased phosphorylation level of JNK plays an important role in SUV-induced skin inflammation. Therefore, inhibiting JNK activity may be a strategy for preventing sunburn.

In recent years, studies have shown that natural compounds can reduce skin inflammation and oxidative stress by regulating classical inflammatory signal transduction pathways (Nichols and Katiyar, 2010; Serafini et al., 2015). On the other hand, natural compounds contain conjugated bonds that can prevent radiation from penetrating the skin and reduce inflammation caused by SUVs (Perez-Sanchez et al., 2018; Leccia et al., 2019.)

As a traditional Chinese medicine, *Coptis* is widely used in the treatment of inflammation, diarrhea, and other diseases (Choi et al., 2013; Wang et al., 2014; Wu et al., 2019). Worenine is one of the active ingredients extracted from *Coptidis rhizoma* (Chen et al., 2010). The biological functions of worenine are unknown. In this study, we intended to investigate the biological function of worenine in inhibiting SUV-induced sunburn. We used SUV resources to imitate SUV irradiation. We determined the pharmacological effects of worenine on skin inflammation using the established model. Furthermore, to elucidate the mechanism, we investigated the effects of worenine on the JNK-ATF2/c-jun signaling pathway, which is involved in the pathological processes of sunburn. Therefore, worenine may be a promising drug candidate for the prevention and treatment of SUV-induced sunburn.

## Materials and Methods

### Cell Culture

HaCaT and JB6 Cl41 cells were purchased from the American Type Culture Collection (ATCC) and passaged in our laboratory for fewer than 6 months after receipt; reauthentication was not needed. The HaCaT cells were cultured with Dulbecco’s minimum essential medium (DMEM) containing 10% fetal bovine serum (FBS). The JB6 Cl41 cells were cultured with a minimum essential medium (MEM) containing 5% fetal bovine serum (FBS).

### Reagents

Traditional Chinese medicine (TCM) biologically active compound standard products containing sweroside standard product (purity > 98%), worenine standard product (purity > 98%), paeonolide standard product (purity > 98%), picroside II standard product (purity > 98%), picroside III standard product (purity > 98%), shikimic acid standard product (purity> 98%), syringic acid standard product (purity > 98%), and paeonifl standard product (purity > 98%) were purchased from Chengdu Refines Biotechnology Co. Ltd. (CN).

### Solar Ultraviolet Irradiation

The SUV resources were purchased from Q-Lab Corporation (Cleveland, OH) and measured using a UV meter. The percentages of UVA and UVB were 92.5% and 7.5%, respectively. Approximately, 40 KJ/m^2^ SUV irradiation was used in the cellular study, and 100 KJ/m^2^ SUV irradiation was used in the animal study. This method was described in detail in our previous study (Wu et al., 2016).

The cells were cultured at 70% confluence and then cultured using serum-free culture media containing the corresponding compounds for 12 h. The cells were irradiated under 40 KJ/m^2^ of SUV for 48 min followed by placement at room temperature for 5 min. Then, the culture supernatant was collected for ELISA detection, and the cells were collected for Western blotting.

### 4,5-Dimethythiazoyl-zyl2,5-Diphenylterrajolium Bromiole Assay

Cells (5 × 10^3^/well) were seeded in 96-well plates and cultured at 37°C and 5% CO_2_ for 24 h. Then, fresh media with different concentrations of the corresponding compounds were added for 24 and 48 h, and cell viability was measured by MTT. Ninety microliters of fresh media with 10 μl of MTT (0.5 mg/ml) was added per well for 4 h, the culture media was discarded, and 150 μl of DMSO was added. The absorbance was measured at OD_490/630_ nm when the crystal was fully dissolved. The concentrations of compounds with cell viability above 80% were used in follow-up experiments.

### Western Blotting

#### Cell Lysate Collection

HaCaT or JB6 Cl41 cells were cultured at 70% confluence and then cultured using serum-free culture media containing the corresponding compounds for 12 h. The cells were irradiated under 40 KJ/m^2^ of SUV for 48 min followed by placement at room temperature for 5 min. Then, the cells were collected for Western blotting. Two hundred microliters of RIPA buffer was then added to disrupt the cells.

#### Mouse Skin Lysate Collection

The mouse skin was ground with liquid nitrogen, sonicated in cold PBS, and centrifuged for 10 min at 12,000 rpm. The supernatant was collected for detection.

### Western Blotting Protocols

Protein content was quantified using the Bradford method. The samples were resolved by 10%–15% SDS-PAGE and transferred onto PVDF membranes (Millipore). After that, the membrane was blocked with 5% fat-free milk or BSA for 20–30 min and washed with TBST, followed by incubation with the indicated primary antibodies overnight at 4°C. The following primary antibodies were used: rabbit anti-JNK (Cell Signaling Technology, 9252), rabbit anti-phospho-JNK (Thr183/Tyr185) (Cell Signaling Technology, 4668), rabbit anti-p38 (Cell Signaling Technology, 2308), rabbit anti-phospho-p38 MAPK (Thr180/Tyr182) (Cell Signaling Technology, 4511), rabbit anti-ATF2 (Cell Signaling Technology, 9226s), rabbit anti-phospho-ATF2 (Cell Signaling Technology, 5112), mouse anti-β-actin (Santa Cruz, SC-47778), rabbit anti-H2AX (Cell Signaling Technology, 7631), rabbit anti-phospho-Histone H2AX (Ser139) (Cell Signaling Technology, 3377s), and mouse anti-GST-tag (Proteintech Group, 66031-1-Ig). The chemiluminescence method (Bio-Rad) was used to detect the signals.

### Enzyme-Linked Immunosorbent Assay

#### Cell Culture Supernatant Collection

Cells were cultured at 70% confluence and then cultured using serum-free culture media containing the corresponding compounds for 12 h. The cells were irradiated under 40 KJ/m^2^ of SUV for 48 min followed by placement at room temperature for 5 min. Then, the cell culture supernatant was collected for detection.

#### Mouse Skin Lysate Collection

The mouse skin was ground with liquid nitrogen, sonicated in cold PBS, and centrifuged for 10 min at 12,000 rpm. The supernatant was collected for detection.

### Enzyme-Linked Immunosorbent Assay Protocols

All reagents were equilibrated at room temperature for 30 min and mixed well before use. The ELISA plate was soaked using 1× washing buffer and dried on absorbent paper. Standard solutions were configured and diluted two-fold to the different standard wells. The cell culture supernatant or mouse skin lysate was added to the sample wells. IL-6 or TNF-α antibody was added to each well and incubated at 37°C for 90 min. After that, the ELISA plate was washed and dried on absorbent paper six times. Streptavidin-HRP working solution was added and incubated at 37C for 30 min. The ELISA plate was washed and dried on absorbent paper six times. Then, 100 μl of TMB was added and incubated at 37°C in the dark for 20 min, followed by the addition of a stop solution to stop the reaction. The absorbance was measured at OD_450_ nm with a microplate reader (Bio-Rad, Kyoto, Japan).

### Kinase Array

A kinase array was used to test the inhibitory effects of worenine on the activities of those predicted target kinases in a commercial kinase library. Worenine was prepared to a 50× final assay concentration in 100% DMSO. This working stock (12.5 μM) was added to the assay well as the first component in the reaction, followed by the panels of kinases available commercially, corresponding substrates, and Mg/[γ-^33^P]ATP mix. After incubation for 40 min at room temperature, the reaction was stopped by the addition of phosphoric acid to a concentration of 0.5%. Then 10 microliters of the reaction was spotted onto a P30 filtermat and washed four times for 4 min in 0.425% phosphoric acid and once in methanol prior to drying and scintillation counting.

In the standard KinaseProfiler service, the blank wells contained all components of the reaction, except the compound of interest. However, DMSO (at a final concentration of 2%) was included in these wells to control for solvent effects. The positive control wells contained all components of the reaction, with a reference inhibitor replacing the compound of interest.

### Protein Prokaryotic Expression and Purification

#### Protein Prokaryotic Expression

pGEX-GST-H2AX and pGEX-GST-JNK were expressed in *Escherichia coli* BL21. Bacteria were grown at 37°C to an absorbance of 0.6–0.8 at 600 nm. After that, 1 mM isopropyl β-d-thiogalactopyranoside (IPTG) was added for 3 h to induce high protein expression. The bacteria were centrifuged at 3,000 rpm for 10 min and then washed with cold 1× PBS three times. The bacterial precipitates were frozen at −80°C and thawed at 37°C three times. The bacterial precipitates were sonicated for 20 min with cold 1× PBS and then centrifuged for 10 min at 12,000 rpm.

#### pGEX-GST-JNK2 Purification

The supernatant was collected and purified with glutathione agarose (Qiagen) overnight at 4°C and then washed with wash buffer. After that, the samples were resolved by 10% SDS-PAGE and visualized by Coomassie brilliant blue staining.

#### pGEX-GST-H2AX Purification

The pellet was resuspended with protein preservation solution (Abcam) and then sonicated. After centrifugation at 12,000 rpm for 15 min, the supernatant and precipitate were separated, resolved by 10% SDS-PAGE, and visualized by Coomassie brilliant blue staining.

### *In Vitro* Kinase Assay

The GST-H2AX fusion protein was obtained through the prokaryotic expression method and used as the substrate. Active JNK2 kinase (0.15 μg) was pre-reacted with worenine at 32°C for 40 min. Then, active JNK2 kinase (0.15 μg) without or with worenine pretreatments was incubated with GST-H2AX (3 μg) in a 30 μl reaction containing 100 nM ATP at 37°C for 2 h. Subsequently, 5× loading buffer was added to the sample, heated at 95°C for 10 min, and analyzed by Western blotting using the indicated antibodies.

### Microscale Thermophoresis

The GST-JNK2 fusion protein was obtained through the prokaryotic expression method and used for the microscale thermophoresis (MST) assay. The worenine solution (2 mM) was used as an initial working solution (solution 1) for the determination of binding force. Then, solution 1 was diluted in multiples of 2 with HEPES buffer (20 mM, pH 7.5). The concentration of solution 16 was 0.14 nM. Next, 869.6 nM JNK2 was added to each working solution, and the equilibrium dissociation constant K_D_ was measured on a microthermograph.

### Animal Study

Seven-week-old male Balb/c mice were purchased from the Animal Experiment Center of Sanxia University (Hubei, China). The mice were divided into the following four groups: vehicle group (CON), SUV irradiation group (SUV), worenine/SUV group (WOR), and paeonol/SUV group (PAE). Paeonol is a drug that can inhibit sunburn and was used as a positive control. The backs of the mice were shaved with hair removal cream 24 h before the experiment. Worenine or paeonol solution was mixed evenly with Carbomer 940 cream to a final concentration of 2 mM. Thirty milligrams of worenine cream (0.07%) or 30 mg paeonol cream (0.035%) was applied to the backs of the mice in the worenine/SUV group or paeonol/SUV group. The backs of mice in the vehicle group and SUV group were smeared with the same amount of Carbomer 940 cream. Three hours later, except for the vehicle group, the mice were fixed with their backs facing the SUV lamps. A 100 KJ/m^2^ SUV was used to irradiate the backs of the mice for 2 h. Twenty-four hours after SUV irradiation, the mice were sacrificed, and the back skin was taken for Western blotting, ELISA, and H&E assays. All mouse experiments were performed in strict accordance with the Institutional Animal Care and Use Committee, Tongji Medical College, Huazhong University of Science and Technology.

SUV irradiation induces skin inflammation through an increase in epidermal thickness and the secretion of inflammatory factors. Compared with the vehicle group, the skin epidermal layer on the backs of the mice in the SUV irradiation group had thickened significantly, and the blood vessels were enlarged. At the same time, more obvious symptoms of edema, epithelial cell proliferation, and immune cell infiltration appeared. The above symptoms indicate that the mouse model was successfully established.

### Hematoxylin and Eosin

The mouse skins were fixed with paraformaldehyde, dehydrated, cleared, and embedded in paraffin. The prepared paraffin sections were deparaffinized with xylene, transferred to distilled water for staining, and then stained with hematoxylin solution. Then, the sections were immersed in 70% and 90% alcohol, dehydrated for 10 min, and stained with ethanol–eosin staining solution. The stained sections were dehydrated with pure ethanol, washed with xylene, covered and sealed with a cover glass, and finally photographed using an Olympus Imaging System Microscope.

### Statistics

The experiments were repeated at least three times, and all data represent the results of triplicate experiments. The data are presented in the form of $\bar{X}$ ± SD, *p < 0.05, **p < 0.01, and ***p < 0.001. Statistical analysis charts were made with Prism 5, and a t-test was used in the statistical analysis.

## Results

### Worenine Inhibits the Increase in p-JNK Induced by Solar Ultraviolet

**FIGURE 1:**
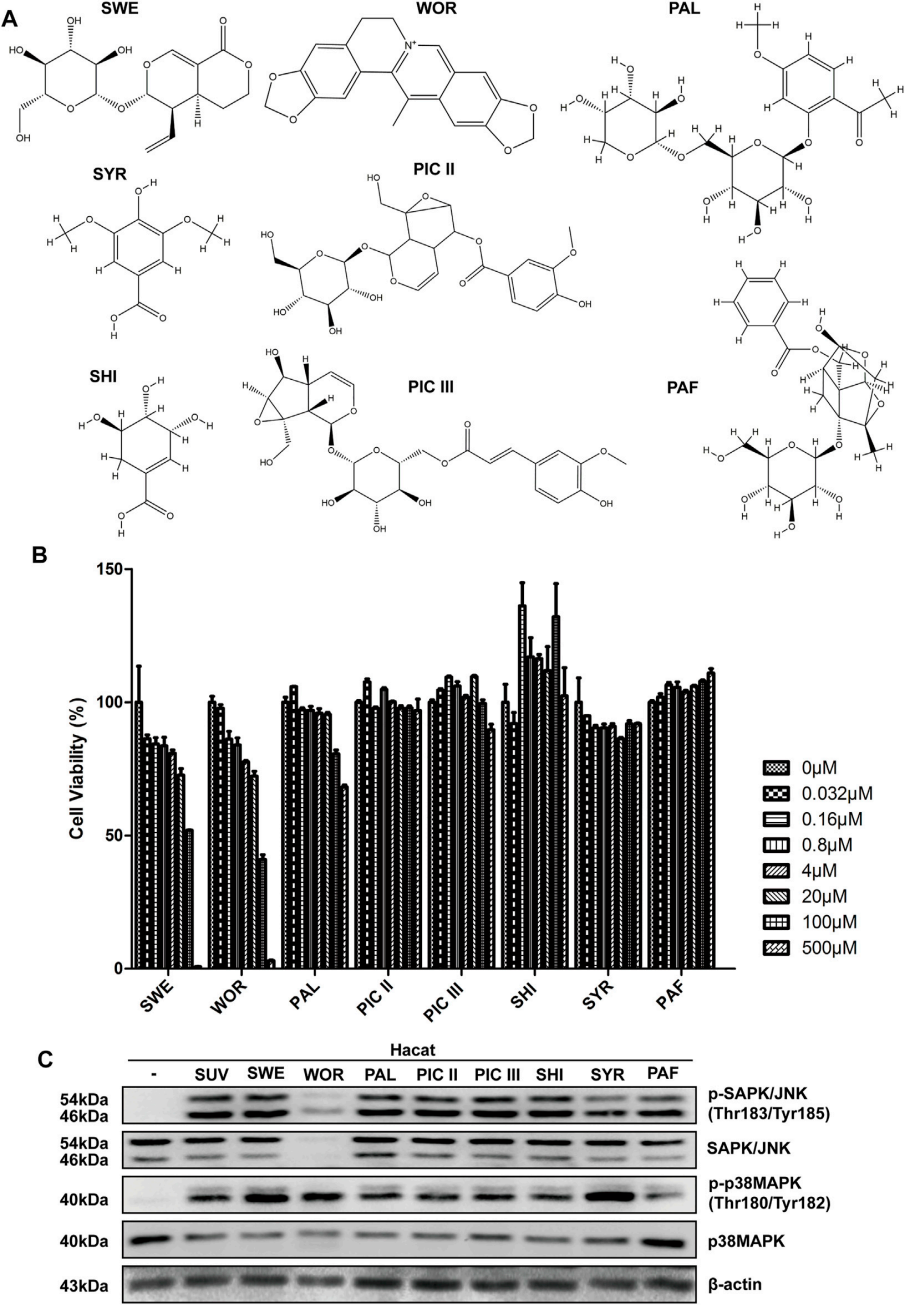
Worenine inhibits the increase of p-JNK induced by SUV. **(A)** The chemical structural formulas of eight kinds of traditional Chinese medicine biologically active compounds. **(B)** HaCaT cells treated with these compounds for 48 h, and cell viability measured by MTT. **(C)** HaCaT cells treated with these compounds for 12 h followed by SUV irradiation. Cells were collected to detect the phosphorylation levels of JNK and p38 by Western blot using the corresponding antibodies.

To explore compounds with therapeutic effects on sunburn, our research team selected several traditional Chinese medicine biologically active compounds from natural Chinese medicinal materials with antioxidant and anti-inflammatory effects. They were sweroside (SWE, from *Lonicera japonica*), worenine (WOR, from *Coptis chinensis*), paeonolide (PAL, from *Paeonia suffruticosa*), picroside II/III (PIC II/III, from *Picrorhiza scrophulariiflora* Pennell), shikimic acid (SHI, from *Illicium verum*), syringic acid (SYR, from *Spirulina*), and paeoniflorin (PAF, from *Paeonia lactiflora* Pallas), and their structures are shown in Figure 1A. The compounds we selected might show inhibitory effects on sunburn. To test whether the compounds show inhibitory effects on sunburn, HaCaT cells were treated with these compounds, and cell viability was measured by MTT. The concentrations of compounds with cell viability above 80% were used in follow-up experiments (Figure 1B). SUV radiation quickly activates MAPK signaling pathways, such as the Erk, JNK, and p38 MAPK signaling pathways (López-Camarillo et al., 2012). The activation of the Erk signaling pathway mainly regulates cell proliferation and survival. The JNK and p38 signaling pathways, which are the two classic signaling pathways associated with inflammation, are activated to produce protective and proapoptotic effects (Wu et al., 2016). To confirm whether these compounds could inhibit the JNK and p38 MAPK signaling pathways in SUV-induced sunburn, the phosphorylation levels of JNK and p38 were detected using Western blotting. The results showed that only worenine inhibited the increase in p-JNK induced by SUV, but it showed no inhibitory effects on p38 phosphorylation (Figure 1C). The increased phosphorylation level of JNK plays an important role in SUV-induced skin inflammation. Therefore, the results showed that worenine might alleviate SUV-induced sunburn by inhibiting JNK.

### Worenine Inhibits the Solar Ultraviolet–Induced JNK-ATF2/c-Jun Signaling Pathway and IL-6 and TNF-α Secretion in Cell Culture

**FIGURE 2:**
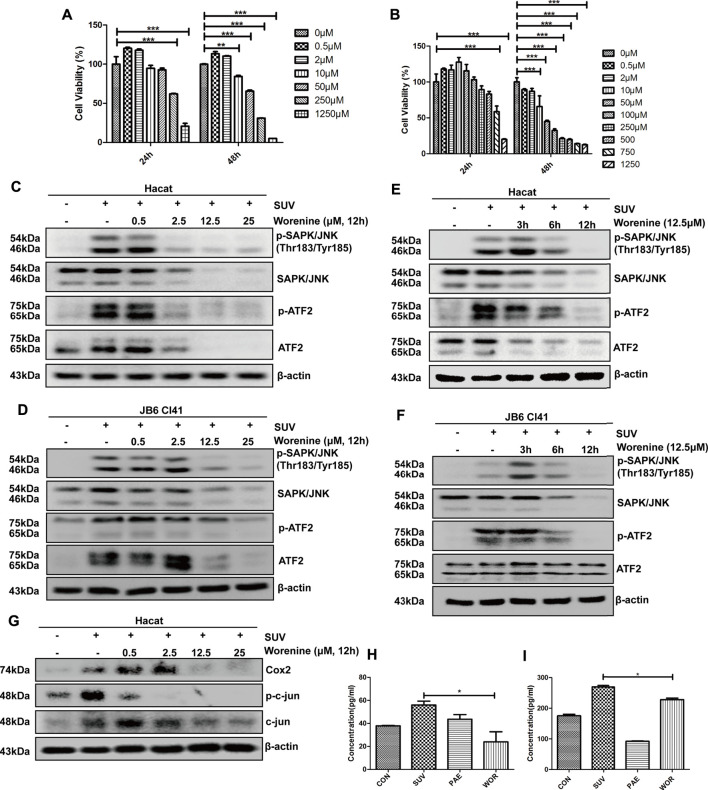
Worenine inhibits SUV-induced JNK-ATF2/c-jun signaling pathway, and IL-6 and TNF-α secretion in cell culture. **(A,B)** Different concentrations of worenine were used to treat HaCaT cells **(A)** and JB6 Cl41 cells **(B)** for 24 and 48 h, respectively, and MTT was used to detect the cell viability. **(C,D)** HaCaT **(C)** and JB6 Cl41 **(D)** cells were treated with different concentrations of worenine for 12 h followed by SUV irradiation, and then collected to detect the JNK-ATF2 signaling pathway by Western blot using indicated antibodies. **(E,F)** HaCaT **(E)** and JB6 Cl41 **(F)** cells were treated with 12.5 μM of worenine for different times before SUV irradiation and then collected to detect the JNK-ATF2 signaling pathway by Western blot using indicated antibodies. **(G)** HaCaT cells were treated with different concentrations of worenine for 12 h before SUV irradiation, and then collected for Western blot assay using indicated antibodies. **(H,I)** HaCaT cells were treated with worenine for 12 h before SUV irradiation, the cell culture supernatant was then collected to detect the secretion of IL-6 **(H)** and TNF-α **(I)** using ELISA assay. Paeonol was used here as a positive control for drug treatment. The data are presented in the form of $\bar{\text{X}}$ ± SD, *p < 0.05, **p < 0.01, ***p < 0.001.

To further demonstrate whether worenine alleviates SUV-induced sunburn by inhibiting JNK, HaCaT and JB6 Cl41 cells were treated with worenine, and cell viability was detected by MTT. The concentration of worenine with cell viability above 80% was chosen for subsequent experiments (Figures 2A,B). To test whether worenine could inhibit the JNK-ATF2 signaling pathway in SUV-induced sunburn, HaCaT and JB6 Cl41 cells were treated with worenine before SUV irradiation and collected to detect the JNK-ATF2 signaling pathway using Western blotting. The results showed that worenine inhibited the activation of the JNK-ATF2 signaling pathway induced by SUV in a dose- (Figures 2C,D) and time-dependent manner (Figures 2E,F).

Similarly, the phosphorylation of c-jun was inhibited by worenine (Figure 2G). AP1 is the transcription factor of IL-6 (An et al., 2003), and we further tested whether worenine could affect the secretion of the pro-inflammatory factor IL-6 in HaCaT cells using ELISA. The results showed that worenine significantly reduced the secretion of IL-6 induced by SUV (Figure 2H). Similar to IL-6, the secretion of TNF-α was also reduced by worenine (Figure 2I).

As the increased secretion of pro-inflammatory factors plays a vital role in SUV-induced skin inflammation, the above results have indicated that worenine could inhibit SUV-induced IL-6 and TNF-α secretion to prevent sunburn in cell culture.

### Worenine Binds With and Inhibits JNK2 Activity *In Vitro*

**FIGURE 3:**
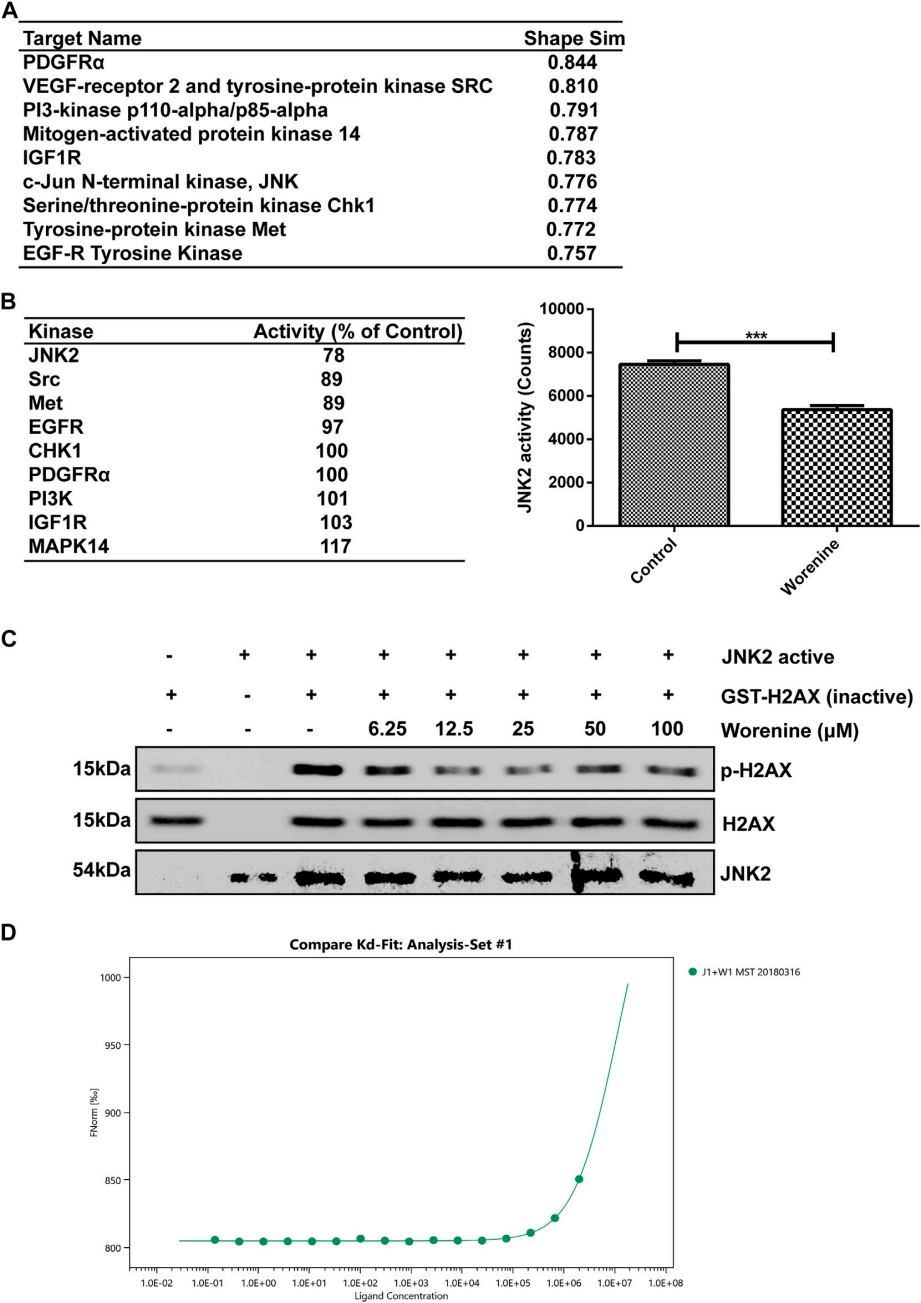
Worenine binds with and inhibits JNK2 activity *in vitro*. **(A)** Bioinformatics predicted some potential targets of worenine. **(B)** 12.5 μM of worenine was used to test its inhibitory effects on the activity of kinases in a commercial kinase library using a kinase array assay. JNK2 activity was analyzed using Prism 5 software. The data are presented in the form of $\bar{\text{X}}$ ± SD, *p < 0.05, **p < 0.01, ***p < 0.001. **(C)** Different concentrations of worenine were used to pretreat active JNK2 kinase at 32°C for 40 min, and then this reaction complex was used as a kinase, the GST-H2AX fusion protein as a substrate for an *in vitro* kinase assay, then the phosphorylation level of H2AX was detected by Western blot. **(D)** Measurement of affinity between worenine and JNK2 by MST in standard treated capillaries and the resulting binding curve are shown. From the resulting binding curve, with a Kd of 116.4 μM.

Next, we explored the target of worenine. First, bioinformatics predicted some potential targets of worenine, among which the probability score of JNK was 0.776 (Figure 3A). Then, a kinase array was used to test the inhibitory effects of worenine on the activities of those predicted targets. The results showed the best inhibitory effects of worenine on JNK2, and 12.5 μM worenine inhibited JNK2 activity by nearly 22% (Figure 3B), indicating that worenine could inhibit the kinase activity of JNK2 *in vitro*. JNK2 phosphorylates H2AX at Ser139 and promotes apoptosis, and the phosphorylation of H2AX can reflect JNK2 activity (Lu et al., 2006). An *in vitro* kinase assay was used to detect the inhibitory effects of worenine on the activities of JNK2. The results showed that worenine pretreatment reduced the phosphorylation level of H2AX in a dose-dependent manner, indicating that worenine inhibited JNK2 activity (Figure 3C). In summary, the above results have indicated that worenine could inhibit JNK2 activity *in vitro* and that JNK2 might be the target of worenine.

Furthermore, an MST assay was used to determine whether there was an interaction between worenine and JNK2. The results showed that worenine bound to JNK2 *in vitro*, with a K_D_ of 116.4 μM (Figure 3D).

The results above indicated that worenine bound with and inhibited JNK2 activity *in vitro.*

### Worenine Inhibits Solar Ultraviolet–Induced Sunburn and JNK Activation in Mouse Model

**FIGURE 4:**
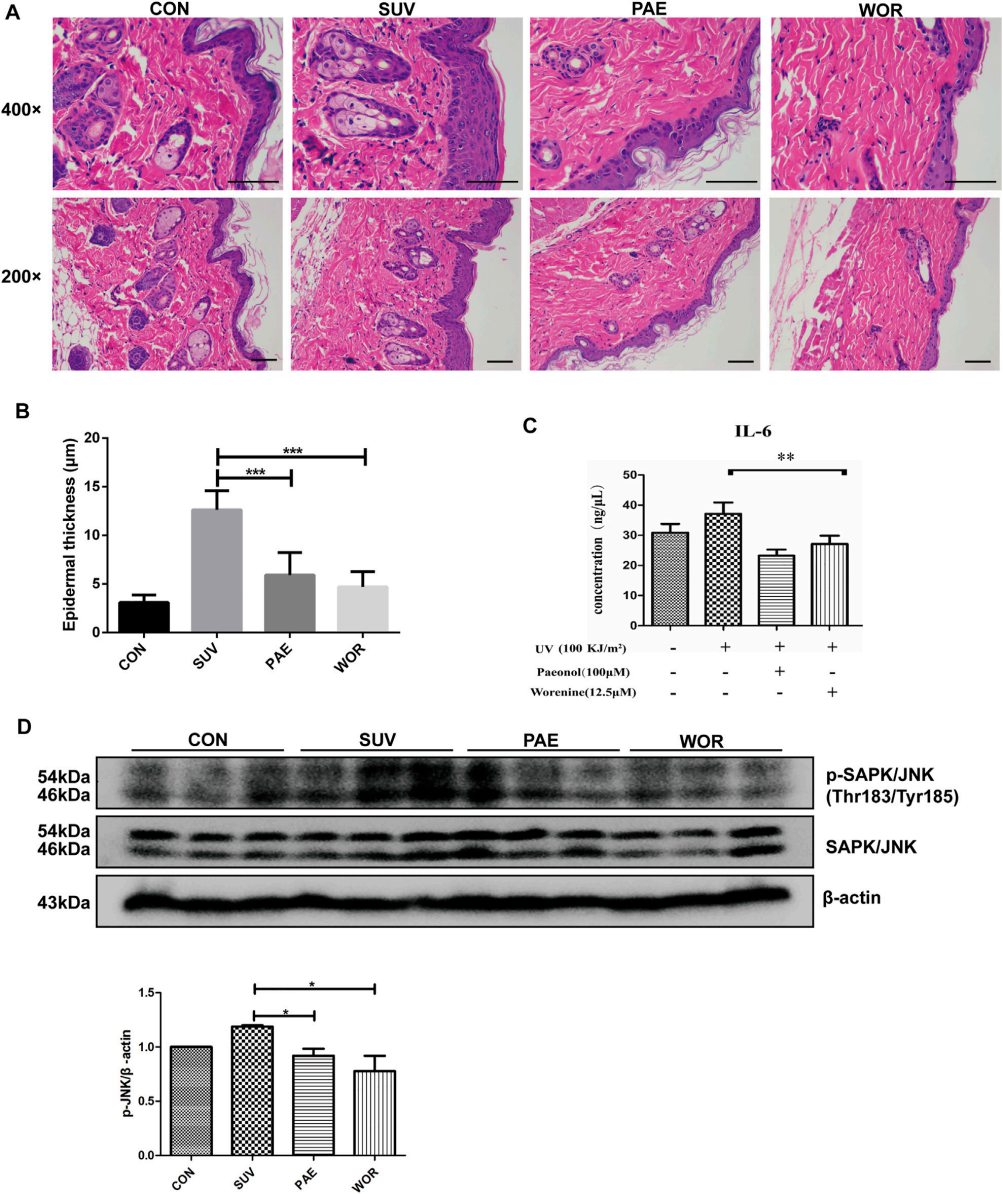
Worenine inhibits SUV-induced sunburn and JNK activation in a mouse model. **(A)** The back skin was taken for H&E staining analysis in the mouse model of SUV-induced sunburn. Scale bar: 10 μm. **(B)** The epidermal thickness of the back skin was analyzed using ImageJ software. The data are presented in the form of $\bar{\text{X}}$ ± SD, *p < 0.05, **p < 0.01, ***p < 0.001. **(C)** The back skin was taken to detect IL-6 secretion using ELISA assay in the mouse model of SUV-induced sunburn. The data are presented in the form of $\bar{\text{X}}$ ± SD, *p < 0.05, **p < 0.01, ***p < 0.001. **(D)** The back skin was taken to detect the phosphorylation level of JNK using Western blot in the mouse model of SUV-induced sunburn. The data are presented in the form of $\bar{\text{X}}$ ± SD, *p < 0.05, **p < 0.01, ***p < 0.001.

SUV irradiation induces skin inflammation with increased epidermal thickness and inflammatory factors (Moore et al., 2013; Birner et al., 2016; Jandova et al., 2021). In addition, the increased phosphorylation level of JNK can reflect SUV-induced skin inflammation (Lu et al., 2022). Finally, we further verified the anti-inflammatory effect and the inhibitory effect of worenine on JNK activity in the sunburn model of Balb/c mice. The H&E staining results showed that compared with the vehicle group, the skin epidermal layer on the backs of the mice in the SUV irradiation group was significantly thickened, and the blood vessels were enlarged. At the same time, more obvious symptoms of edema, epithelial cell proliferation, and immune cell infiltration appeared. The above symptoms indicate that the mouse model was successfully established. Compared with the SUV irradiation group, the skin epidermal layer on the backs of the mice in the worenine and paeonol pretreatment groups was significantly thinner, the blood vessels became smaller, and the symptoms of edema, epithelial cell proliferation, and immune cell infiltration were significantly weakened (Figures 4A,B). The results have indicated that worenine could inhibit SUV-induced sunburn in a mouse model.

ELISA results have shown that compared to the vehicle group, IL-6 secretion in the skin of the mice in the SUV irradiation group was increased. Compared with the SUV irradiation group, IL-6 secretion in the skin of the mice in the worenine and paeonol pretreatment groups was significantly reduced (Figure 4C). The results indicated that worenine could inhibit SUV-induced IL-6 secretion in a mouse model.

The Western blotting results showed that compared to the vehicle group, the phosphorylation level of JNK in the skin of the mice in the SUV irradiation group was significantly increased. Compared to the SUV irradiation group, the phosphorylation level of JNK in the back skin of the mice in the worenine and paeonol pretreatment groups was significantly reduced (Figure 4D), indicating that worenine could inhibit SUV-induced activation of JNK in a mouse model.

Overall, the above results indicated that worenine could inhibit SUV-induced sunburn, IL-6 and TNF-α secretion, and JNK activation in a mouse model.

## Discussion

Excessive solar ultraviolet (SUV) radiation often causes a series of skin lesions, such as hyperpigmentation, dermatitis, photoaging, and even skin cancer. In the pathological processes of SUV-induced sunburn, JNK is activated, specifically manifesting as the increased phosphorylation of JNK. Activated JNK further phosphorylates and activates its downstream transcription factors, leading to increased secretion of inflammatory cytokines and aggravation of the inflammatory response (Blaser et al., 2016; Zeke et al., 2016). Therefore, inhibiting JNK can be a therapeutic strategy for sunburn. Inhibiting JNK activity and interfering with JNK signaling pathways can alleviate inflammation, so it provides new treatment strategies for SUV-induced photoaging and skin cancer.

As there are varying degrees of side effects and poor specificity in JNK inhibitors, it is necessary to develop new JNK inhibitors. In this study, bioinformatics prediction have shown that worenine was a potential inhibitor of JNK. The MST assay confirmed that worenine bound *in vitro* to JNK2, one of the JNK family subtypes. Kinase array and *in vitro* kinase assays were used to confirm that worenine inhibited JNK2 activity *in vitro*. Then, worenine was confirmed to inhibit the JNK-ATF2/c-jun signaling pathway in cell culture and animal models of sunburn induced by SUVs. Therefore, worenine was expected to become an inhibitor targeting JNK2, which requires further exploration. For example, the effects of JNK phosphorylation *in vitro* and *in vivo* were different. *In vivo*, SUV-induced p-JNK levels were attenuated by pretreatment with worenine on the skin. *In vitro*, both total SAPK/JNK and p-JNK were lowered by worenine with SUV. *In vitro*, total SAPK/JNK was markedly decreased by treatment with worenine. Two reasons may account for the difference. On one hand, the dose of worenine *in vivo* study was too low to affect total SAPK/JNK, but only pJNK. In our mouse model, the dose of worenine was 21 μg/cm^2^, which was lower than the international standardized sunscreen dose of 2 mg/cm^2^ (Young et al., 2017). On the other hand, the possibility of JNK inhibition leads to its degradation, which may be caused by the autophagy–lysosome pathway or ubiquitin-proteasome proteolytic pathway. Our next study will attempt to confirm the direct pathway involved in the treatment.

In recent years, the development of drugs for SUV-induced solar dermatitis has focused more on natural medicines because they are natural and inexpensive (Choi et al., 2013). *C. chinensis* exerts anti-inflammatory and antioxidation effects (Zhou et al., 2016). Worenine is a biologically active compound extracted from *C. chinensis*, but its biological function is unknown. As a natural drug, worenine has unsaturated bonds in its structure, conforming to the structural characteristics of UV absorption. In this study, through bioinformatic prediction and preliminary Western blot screening, we found that worenine was a potential drug candidate for inhibiting sunburn. The *in vitro* study confirmed that worenine inhibited JNK2 activity. In cell culture and animal models that simulated SUV-induced sunburn, we found that worenine exerted a significant anti-inflammatory effect. In this study, we could not exclude the role of worenine in absorbing UV light to protect our skin against SUV, and the mechanism by which worenine played a more important role requires more in-depth research. Maybe worenine protects against SUV in two ways. On the one hand, worenine absorbs SUV radiation because of its conjugated double bonds. However, worenine inhibits JNK2 to inhibit sunburn. In this study, worenine was applied before SUV irradiation, and whether the application of worenine after SUV irradiation is effective needs further research. Research on its inhibitory effect on sunburn is undoubtedly good news for the pharmaceutical industry and cosmetics industry, and it is also a benefit to people who are exposed to SUVs. There is a broad application market that can bring huge economic benefits, and worenine is expected to become a dermatitis prevention and treatment drug with great prospects and potential.

## Conclusion

In summary, our study screened for worenine as a potential drug candidate for inhibiting JNK2 activity to inhibit sunburn. Worenine bound to and inhibited the activity of JNK2, suppressed the JNK-ATF2 signaling pathway, and inhibited the secretion of IL-6 and TNF-α, thereby preventing SUV-induced sunburn. Therefore, worenine might be a promising drug candidate for the prevention and treatment of SUV-induced sunburn.

## Data Availability

The datasets presented in this study can be found in online repositories. The names of the repository/repositories and accession number(s) can be found at: http://www.wwpdb.org/3E7O, and https://pubchem.ncbi.nlm.nih.gov/compound/20055073.

## Abbreviations

JNK/SAPK, stress-activated protein kinases; PAF, paeoniflorin; PAL, paeonolide; PIC II/III, picroside II/III; RNS, reactive nitrogen species; ROS, reactive oxygen species; SHI, Shikimic acid; SUV, solar ultraviolet; SWE, sweroside; SYR, syringic acid; TCM, traditional Chinese medicine; WOR, worenine.
